# Improving read mapping using additional prefix grams

**DOI:** 10.1186/1471-2105-15-42

**Published:** 2014-02-05

**Authors:** Jongik Kim, Chen Li, Xiaohui Xie

**Affiliations:** 1 Division of Computer Science & Engineering, Chonbuk National University, Jeonju, Republic of Korea; 2 Department of Computer Science, University of California, Irvine, USA

**Keywords:** Next-generation sequencing, Read alignment, All mapper, Additional prefix *q*-gram, Hobbes2

## Abstract

**Background:**

Next-generation sequencing (NGS) enables rapid production of billions of bases at a relatively low cost. Mapping reads from next-generation sequencers to a given reference genome is an important first step in many sequencing applications. Popular read mappers, such as Bowtie and BWA, are optimized to return top one or a few candidate locations of each read. However, identifying all mapping locations of each read, instead of just one or a few, is also important in some sequencing applications such as ChIP-seq for discovering binding sites in repeat regions, and RNA-seq for transcript abundance estimation.

**Results:**

Here we present Hobbes2, a software package designed for fast and accurate alignment of NGS reads and specialized in identifying all mapping locations of each read. Hobbes2 efficiently identifies all mapping locations of reads using a novel technique that utilizes additional prefix *q*-grams to improve filtering. We extensively compare Hobbes2 with state-of-the-art read mappers, and show that Hobbes2 can be an order of magnitude faster than other read mappers while consuming less memory space and achieving similar accuracy.

**Conclusions:**

We propose Hobbes2 to improve the accuracy of read mapping, specialized in identifying all mapping locations of each read. Hobbes2 is implemented in C++, and the source code is freely available for download at http://hobbes.ics.uci.edu.

## Background

DNA sequencing has become an indispensable tool for basic biomedical research, understanding disease mechanisms, and developing new and personalized treatments. Recent advances in next-generation sequencing (NGS) technologies, such as those from Illumina and Life Technologies, have enabled the rapid production of billions of bases at relatively low cost. However, the reads returned by NGS sequencers are usually short (in the range of 35 to 150 bps), and it is left to computational algorithms to extract information from these reads.

In many applications, mapping reads to a given reference genome sequence is an important first step in analysis of sequencing data. Popular read mapping programs, such as Bowtie [1], Bowtie2 [2], and BWA [3], aim at identifying one or a few top mapping locations for each read. This mapping strategy works well for many applications, and leads to a significant improvement in mapping speed compared to programs aiming at identifying all candidate locations. However, in many applications, it is often more desirable to identify all candidate locations of reads. (We will call programs that can identify all candidate locations *all mappers*). For instance, in ChIP-seq experiments, many binding sites are located in the repeat regions of the genomes, and therefore, using read mappers returning only one or a few mapping locations might miss many binding peaks located within these repeat regions [4]. In RNA-seq transcript abundance quantification, due to the presence of multiple transcript isoforms caused by alternative splicing, it is critical for a read mapper to return all possible mapping locations. Otherwise, the accuracy of the transcript abundance estimation can be significantly compromised [5,6].

As the sequencing technology is progressing toward producing longer reads, it is also very important to support insertion/deletion (indel) errors, which are caused by sequencing errors and/or genetic variations. Hobbes [7] is a software package proposed to identify all mapping locations of a read. It generates candidate locations efficiently using inverted lists of non-overlapping *q*-grams with the help of bit vectors. In the presence of indel errors, however, bit vectors may filter out true locations, which negatively affects the accuracy of the results. Moreover, Hobbes may require a large amount of memory space for bit vectors. Recently developed all mappers, such as RazerS3 [8] and Masai [9], have focused on supporting indel errors and improved the performance in terms of accuracy and mapping time. RazerS3 can generate accurate mapping results by controlling mapping sensitivity based on its error-estimation technique. However, it requires a lot of time to produce high-quality results. Masai reduces mapping time significantly by building an index on input reads and simultaneously generating candidate locations for multiple reads. However, Masai does not support multi-threading since it builds an index on input reads and it is not straightforward to split input reads so that they can be processed by multiple threads.

In this paper, we present Hobbes2, a software package designed to return all mapping locations of long reads (e.g., 100bp or 150bp) containing indel errors as well as mismatch errors. Hobbes2 is built on top of Hobbes but significantly improves the performance in all aspects. Instead of using bit vectors, Hobbes2 makes use of another inverted list of an additional *q*-gram to filter out false positives during the generation of candidate locations. The filtering based on the additional *q*-gram captures all true locations while we can produce substantially fewer candidate locations. By eliminating bit vectors from memory, this approach also greatly saves memory consumption. Hobbes2 aligns reads one by one and naturally scales well in a multi-threaded environment. Because read mappers map a tremendous number of reads, good multi-thread support is extremely important in read alignment. Through experimental comparisons, we show that Hobbes2 is an order of magnitude faster than the best all mappers, RazerS3 and Masai, while consuming less memory space and achieving a similar accuracy.

In the following sections, we briefly describe existing gram-based approaches to solve the read mapping problem and introduce our approach with analysis. Then, we present how to handle indel errors with the proposed approach. We finally discuss the implementation issues to integrate the proposed technique with the existing Hobbes package.

## Methods

After we summarize *q*-gram-based approaches for mapping reads to a given reference genome sequence, we propose a filtering technique using an additional prefix *q*-gram. We first restrict our discussion to the read-mapping problem with mismatch errors only. Then we explain how to extend the proposed technique to support indel errors in a separate section.

### Generating candidate locations using *q*-grams

A *q*-gram of a genome sequence *s* is a subsequence of *s* of length *q*. The set of locationally overlapping *q*-grams of *s*, which is denoted by *G*(*s*), is obtained by sliding a window of length *q* over the bases of *s*. For example, the overlapping 3-gram set of a sequence *s* = ACCTACCT is *G*(*s*)={ACC,CCT,CTA,TAC,ACC,CCT}. Note that we use an ordered multiset for *q*-grams, where the same *q*-grams are distinguished by their locations in a sequence. Because a base of a sequence is included in at most *q* overlapping *q*-grams of the sequence, a substitution of one base modifies at most *q* overlapping *q*-grams of a sequence. Therefore, if the maximum allowed mismatch errors between two sequences of *r* and *s* are *k* bases, they should share at least the following number of common overlapping *q*-grams (this technique is known as count filtering [10]).

(1) T=max{|G(r)|,|G(s)|}−k·q.

As we look for a genome subsequence *s* whose length is the same as a read *r*, we can simplify Equation 1 as follows (because |*G*(*r*)|=|*G*(*s*)|).

(2) T=|G(r)|−k·q=|r|−q+1−k·q.

Many techniques generate candidate locations using inverted-list structures of overlapping *q*-grams of a reference genome sequence. An inverted list of a *q*-gram *g*, denoted by *I*(*g*), is a list of locations within a genome sequence where the *q*-gram occurs. For instance, the inverted list of 3-gram CCT in our previous example is *I*(CCT)={1,4} because CCT occurs at locations 1 and 4 in the sequence ACCTACCT. To map *q*-grams into their corresponding inverted lists, an inverted index is built on overlapping *q*-grams of a genome sequence. Given a read *r* and a Hamming distance threshold *k* (or a maximum number of allowed mismatches *k*), we can generate candidate locations using an inverted index of a genome sequence as follows. First, we decompose *r* into overlapping *q*-grams. For each *q*-gram *g* in *G*(*r*) and its relative location *l* in *r*, we retrieve an inverted list *I*(*g*) by looking up the inverted index with the search key *g*. Because *I*(*g*) contains starting locations of the *q*-gram *g* in the genome sequence, we need to modify *I*(*g*) by subtracting *l* from each element in *I*(*g*) to find the locations of genome subsequences containing *g*. We call a modified inverted list a *normalized inverted list* and denote it by *I*^*n*^(*g*). We finally select those locations as candidates that appear in at least *T* normalized inverted lists.

**Figure 1:**
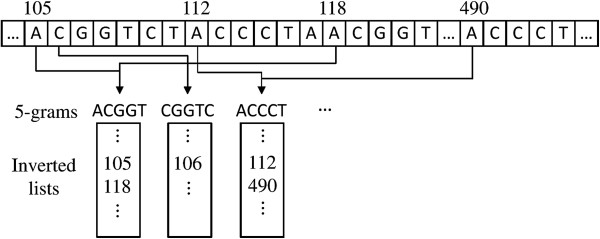
**Excerpt of a reference sequence and a portion of its 5-gram inverted index.** The inverted lists of the 5-grams ACGGT, CGGTC, and ACCCT are shown, each containing a sorted list of locations in the reference sequence where the respective 5-gram appears.

Figure 1 shows an example of a reference genome sequence and its 5-gram inverted index, which is taken from ([7]). To map a read *r* = ACGGTCTTCCCTACGGT with Hamming distance threshold *k*=2 and *T*=17−5+1−2·5=3, we first look up the read’s 5-grams in the inverted index. Notice that only the grams ACGGT and CGGTC (underlined in the read) are present in the index. We traverse their inverted lists, and normalize each element relative to the location of the corresponding gram in the read. For example, the 5-gram CGGTC appears at location 1 in the read, so the relative location of the element on CGGTC’s inverted list is 106−1=105.

In this way, we can count how many times the read’s grams are contained in the subsequence of the reference sequence starting at a fixed location (location 105, in this example). The gram ACGGT appears twice in the read, and we treat each occurrence as a separate list. Its appearance at location 0 yields a normalized list of {105−0=105,118−0=118}, and a normalized list {105−13=92,118−13=105} for location 13. Next, we count the number of occurrences of each element on the normalized lists. The locations 92 and 118 are pruned according to the count filtering, because their number of occurrences do not meet the lower bound of *T*=3. Location 105 has a count of 3, and therefore it is a candidate answer whose Hamming distance to the read still needs to be computed.

In most techniques, the naïve count filtering method is not directly used to generate candidates because it requires scanning all inverted lists of overlapping *q*-grams in a read. To map a 100bp read using an 11-gram inverted index, for example, we need to scan 100−11+1=90 inverted lists. Moreover, some inverted lists are usually very long and this method would incur prohibitive scanning costs. Instead, a simple variation of the count filtering known as the prefix filtering [11] is widely used for generating candidates. Because a candidate genome subsequence *s* needs to contain *Tq*-grams of a read *r* according to the count filtering, *s* must contain at least one *q*-gram among |*G*(*r*)|−(*T*−1)=*k*·*q*+1*q*-grams in *G*(*r*). Given an inverted index of a genome sequence, we retrieve and normalize inverted lists of *k*·*q*+1*q*-grams in *G*(*r*) and then generate candidates by taking the union of locations in the normalized inverted lists. To minimize the number of candidates, we sort *q*-grams in *G*(*r*) by their frequencies in the reference genome and take *k*·*q*+1 low frequency *q*-grams (which are called prefix *q*-grams).

Recent techniques [7,12,13] have focused on deriving a tight lower bound of the number of prefix *q*-grams. The basic idea behind these techniques is to use non-overlapping *q*-grams [14] of a read. If we use non-overlapping *q*-grams, a substitution of one base of a read affects only one *q*-gram. Hence, if a genome subsequence *s* is different from a read *r* within *k* mismatches, the overlapping *q*-gram set of *s* will contain at least one non-overlapping *q*-gram among *k*+1 non-overlapping *q*-grams of *r*. Based on the observation, we can generate candidates as follows. We first select *k*+1 non-overlapping *q*-grams in *G*(*r*). From the inverted index of overlapping *q*-grams of a reference genome sequence, we then retrieve inverted lists of the selected *q*-grams and normalize them. We finally produce candidates by taking the union of locations in the normalized inverted lists. Existing techniques use the sum of frequencies of *q*-grams to estimate the union size of inverted lists. Hobbes proposed a dynamic programming algorithm to select prefix *q*-grams based on the following recurrence so as to minimize the sum of frequencies of selected *q*-grams [7].

(3) $$M(i,j)=\text{min}\;\{\begin{array}{l} M(i,j-1)\\ M(i-1,j)+L[j+(i-1)\cdot q]\mathrm{.len}, \end{array}$$

where *i*≤*k*+1,1≤*j*≤|*G*(*r*)|−*k*·*q*, *L*[ *n*].*len* is the length of the inverted list of the *n*^*th*^*q*-gram in *G*(*r*), and *M*(*i*,*j*) is a lower bound on the sum of the lengths of the inverted lists of *i* non-overlapping grams starting from a location no greater than *j*+(*i*−1)·*q*. In Equation 3, *M*(0,*j*) is initialized to zero and *M*(*i*,0) is initialized to infinity and the goal is to compute *M*(*k*+1,|*G*(*r*)|−*k*·*q*).

### Exploiting an additional prefix *q*-gram of a read

Despite of the effort of the recent work, the number of candidates generated by using optimal prefix selection is still too large to refine and/or verify directly. Thus, it is important to further filter out false positives while generating candidates. Hobbes attaches a bit vector to each element in an inverted list and makes use of bit vectors to remove false positives while generating candidates. However, bit vectors greatly increase the size of an inverted index and thus consume a lot of memory space. In this paper, we propose a powerful and memory efficient filtering method. The proposed method does not require additional memory space while it can still filter out more false positives than bit vectors. Our technique is based on the following lemma.

#### **Lemma1** (Additional prefix)

Given an inverted index of a genome sequence and a read with a Hamming distance threshold *k*, suppose we select *k*+2 non-overlapping *q*-grams from the read. If we retrieve inverted lists of the selected *k*+2*q*-grams from the index, we can select those locations as candidates that come from at least two normalized inverted lists.

The intuition of the lemma is that the set of overlapping *q*-grams in a candidate genome subsequence *s* must contain at least 2 *q*-grams among *k*+2 non-overlapping *q*-grams in a read *r*, because otherwise the difference between *r* and *s* would be larger than *k* bases. We analyze the lemma more precisely using an example as follows. Assume a Hamming distance threshold *k* is 1 and we select *k*+2 non-overlapping *q*-grams *S*={*g*_1_,*g*_2_,*g*_3_} from a read. If we enumerate all possible subsets of S whose cardinality is *k*+1=2, we obtain three subsets, *S*_1_={*g*_1_,*g*_2_}, *S*_2_={*g*_1_,*g*_3_}, and *S*_3_={*g*_2_,*g*_3_}. As described in the previous section, we can generate candidates using any of three subsets. Let *C*(*S*_*i*_) be $\cup_{g\in S_{i}}I^{n}(\;g)$, the set of candidates generated using a subset *S*_*i*_ of *S*. If a candidate location is a true mapping, it should be contained in all of *C*(*S*_1_), *C*(*S*_2_), and *C*(*S*_3_). Therefore, we can generate refined candidates by taking the intersection of *C*(*S*_1_), *C*(*S*_2_), and *C*(*S*_3_). According to this observation, we formulate the set of candidates as

$$(I^{n}(\;\;g_{1})\cup I^{n}(\;\;g_{2}))\cap (I^{n}(\;\;g_{1})\cup I^{n}(\;\;g_{3}))\cap {(}^{n}(\;\;g_{2})\cup I^{n}(\;\;g_{3})).$$

**Figure 2:**
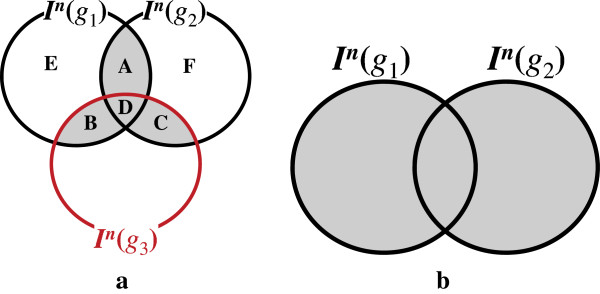
**Filtering effect of an additional prefix*q*-gram.** Gray-scaled areas indicate candidates. **(a)** An additional prefix *q*-gram *g*_3_ plays an important role of filtering out a number of false positives in *E* and *F*. **(b)** If we use *k*+1=2*q*-grams, *g*_1_ and *g*_2_, much more candidates are generated.

Using the distributive, associative, absorption, and idempotent properties of sets, we can rewrite the formula to

$$(I^{n}(\;\;g_{1})\cap I^{n}(\;\;g_{2}))\cup (I^{n}(\;\;g_{1})\cap I^{n}(\;\;g_{3}))\cup (I^{n}(\;\;g_{2})\cap I^{n}(\;\;g_{3})),$$

which is illustrated in a diagram in Figure 2(a). If we compare it with the candidate set generated by *k*+1 prefix *q*-grams *g*_1_ and *g*_2_, which is depicted in Figure 2(b), we can see that an additional prefix *q*-gram *g*_3_ plays a significant role of filtering out false positives.

Given inverted lists of *k*+2 prefix *q*-grams, in general, we can generate candidates by taking the union of pairwise intersections of the inverted lists. That is, each inverted list is intersected *k*+1 times. However, we do not need to scan each inverted list *k*+1 times to generate candidates. Instead, we can use an algorithm that merges all inverted lists by scanning each of them once and selects those locations that appears at least 2 times according to Lemma 1 [see Additional file 1 for the candidate generation algorithm].

### Supporting insertions and deletions

If we use an edit distance threshold (i.e., we allow not only substitutions but also insertions and deletions of bases) for mapping a read, indels introduce two potential problems to the above described technique. In this section, we discuss these two potential problems and describe how to fix them. The first potential problem is caused by insertions or deletions occurred between two matched *q*-grams. In the proposed technique, a candidate genome subsequence *s* needs to contain at least two *q*-grams in a read *r*, where each of which must appear at the same location in both *r* and *s*. In case of edit distance constraints, however, the proposed technique could filter out a valid candidate *s*, since indels between two matched *q*-grams make the locations of the *q*-grams in *r* different from those in *s*.

**Figure 3:**
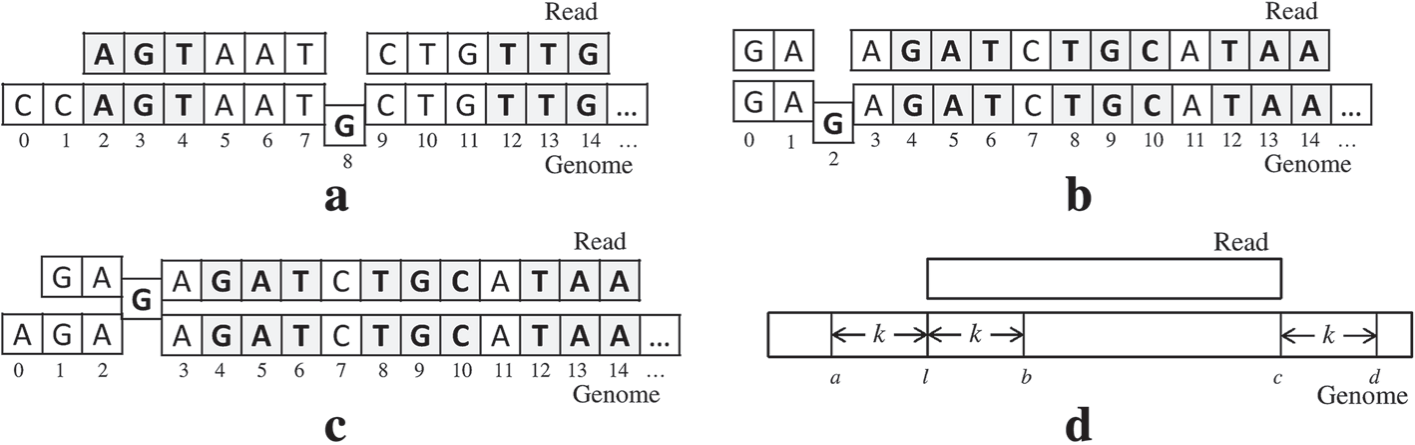
**Problems caused by indels.(a)** Indels occurring between two matched *q*-grams **(b)** Deletions occurring before any matched *q*-grams. **(c)** Insertions occurring before any matched *q*-grams. **(d)** Verification windows of a semi-global alignment algorithm.

For example, consider a genome sequence *S*_*g*_=CCAGTAATGCTGTTG… and a read *r*=AGTAATCTGTTG. Given an edit threshold *k*=1, assume that we select *k*+2=3 non-overlapping tri-grams of *g*_1_=AGT, *g*_2_=ATC, and *g*_3_=TTG in *G*(*r*) (underlined in the read) as the prefix *q*-grams. For *g*_1_, we obtain location 2 of *S*_*g*_ since *g*_1_ appears at location 0 in *r* and at location 2 in *S*_*g*_. For *g*_2_, we cannot find a matched *q*-gram in *S*_*g*_. Finally, for *g*_3_, we get location 3 of *S*_*g*_ since *g*_3_ appears at location 10 in *r* and at location 13 in *S*_*g*_. Because there is no location that appears at least twice, we filter out both locations of 2 and 3. However, the edit distance between the read and the subsequence of *S*_*g*_ starting at location 2 is 1 and we should be able to return location 2 of *S*_*g*_ as a mapping location. The problem is caused by the underlined base G in *S*_*g*_, which is located between two matched grams AGT and TTG as depicted in Figure 3(a).

To fix this problem, we need to allow gaps between two matched *q*-grams up to the edit distance threshold. That is, we treat two locations appearing only once as candidate locations if their difference is within the edit distance threshold. In our example, since the difference between the locations 2 and 3 is within the edit distance threshold 1, we generate both of the locations 2 and 3 as candidates.

The second problem is caused by indels occurring before any locations of matched prefix *q*-grams. If there are *d* deletions of bases in a reference sequence before the matched *q*-grams, we need to consider a subsequence starting at *l*−*d*, where *l* is a candidate location calculated from the matched *q*-grams. For example, consider a genome sequence *S*_*g*_=GAGAGATCTGCATAA… and a read *r*=GAAGATCTGCATAA, where three underlined tri-grams GAT, TGC, and TAA in *G*(*r*) are selected as the prefix grams for an edit distance 1. As our technique returns location 1 in *S*_*g*_ for all the three *q*-grams, we use the location as a candidate and verify the genome subsequence *s* starting at the location. Because the edit distance between *r* and *s* is 2, we do not have a mapping of the read *r*. However, if we consider the subsequence starting from location 0 of *S*_*g*_, we should be able to return the location 0 as a mapping location because the edit distance is 1 as depicted in Figure 3(b). This problem is caused by the deletion of the underlined base G from *S*_*g*_, which is located before the three matched grams GAT, TGC, and TTA.

By contrast, if there are *i* insertions of bases in a reference sequence before any locations of matched prefix *q*-grams, we need to consider a subsequence starting at location *l*+*i*, where *l* is a candidate location. For example, consider a genome sequence *S*_*g*_=AGAAGATCTGCATAA… and a read *r*=GAGAGATCTGCATAA, where three underlined tri-grams are matched for an edit distance 1. Although location 0, which is calculated from the matched tri-grams, does not satisfy the edit distance threshold, we should be able to return location 1 as a mapping location as depicted in Figure 3(c). The underlined base G in *r* (or the insertion of the G into *S*_*g*_) causes this problem.

Therefore, given an edit distance threshold *k* and a candidate starting location *l*, a potential match can start at any location between *l*−*k* and *l*+*k*. Similarly, indels can also occur after matched prefix *q*-grams. Given a candidate ending location *c*, a potential match can end at any location between *c*−*k* and *c*+*k*. So altogether, we need to consider a verification window from *l*−*k* to *c*+*k* to find all potential matches (Figure 3(d)). However, because the verification time based on sequence alignment is proportional to the size of the verification window, enlarging the window at both ends by *k* is computationally expensive. In Hobbes2, we adopt the following heuristic to improve the mapping speed: We first use the verification window [ *l*,*c*+*k*] (Figure 3(d)), and run a semi-global banded alignment algorithm to identify all potential matches located within this window. If this verification window yields no matches, we then consider the verification window [ *l*−*k*,*c*]. This approach could potentially miss some true mappings that start before *l* and at the same time end after *c*. However, empirically we found that those mappings are relatively rare and do not significantly impact the accuracy of our algorithm.

### Implementation details

As Hobbes2 uses an additional prefix *q*-gram instead of bit vectors, it can significantly improve the performance and substantially reduce memory consumption. In this section, we describe how Hobbes2 was implemented on top of Hobbes. Other details and optimization techniques for implementation that are not presented here are the same as those in Hobbes.

Hobbes2 builds an inverted index in the same way that Hobbes does. That is, each element in an inverted list contains a bit vector. However, Hobbes2 loads inverted lists without bit vectors into the memory if it determines that a read has at least *k*+2 non-overlapping *q*-grams, where *k* is a distance threshold. In this case, Hobbes2 filters out false positives using an additional *q*-gram while it generates candidates. If the number of *q*-grams contained in a read is less than *k*+2, Hobbes2 loads both inverted lists and bit vectors into memory. Hobbes2 assumes fixed length input reads and calculate the number of non-overlapping *q*-grams using the length of the first input read. For variable length reads, it also safely maps each read but it does not filter out false positives for those reads that have not enough *q*-grams.

Obviously, the predefined gram length is important for the usability of the proposed filtering technique since it determines the number of *q*-grams in a read. If we increase the gram length, there could be fewer locations in a genome sequence containing the gram, causing the inverted lists to be shorter. Thus, it may decrease the time for scanning inverted lists and produce fewer candidate locations. On the other hand, the size of a hash table for grams in an inverted index becomes very large. If we increase the gram length by 1, the size of the hash table increases by up to 4 times since we have four distinct bases of A, C, G, and T.

Thus, index lookup time for a gram may be the bottleneck of read mapping as the size of the hash table becomes larger. We found that the mapping speed with an 11-gram inverted index was the best when we mapped 100bp reads on HG18 genome sequence [see Additional file 1: Figure S1 for experimental results on gram length]. Based on the experiments, Hobbes2 uses 11-grams and thus it can always find enough grams for a 100bp read with up to 7 errors.

## Results and discussion

### Experimental setup

We implemented Hobbes2 in C++, and compiled it with GCC 4.4.3. All experiments were run on a machine with 94 GB of RAM, and dual Intel Xeons X5670 (12 cores and 24 threads total) at 2.93 GHz, running a 64-bit Ubuntu OS. We performed experiments to examine all mapping capabilities of Hobbes2. We focused on edit distance constraints, and all experiments were performed with the edit distance threshold set to be 5 [see Additional file 1: Table S1 for the experimental results with Hamming distance constraints]. Hobbes2 also has an optional *m*-mapping mode, which returns the results of only those reads whose maximum number of distinct mapping locations is less than or equal to a given threshold *m*. We reported the experimental results on the *m*-mapping mode in Additional file 1: Table S2.

We thoroughly compared Hobbes2 with three state-of-the-art all mappers - Hobbes, RazerS3, and Masai, and three other popular read mappers - GEM [15], BWA and Bowtie2. We did not include other all mappers (such as SOAP2 [16], SHRiMP2 [17], mrsFAST [18], and mrFAST-CO [19]) in our comparison as it has been shown previously that these all mappers do not perform as well as RazerS3, Masai, and/or Hobbes. We configured read mappers to output results in the SAM format with cigar strings [see Section S3 in Additional file 1 for the details of the configuration of each read mapper].

### Index construction and memory footprint

For each reference genome, we built an inverted index of overlapping *q*-grams on the reference genome. By default, Hobbes2 uses 16-bit vectors, resulting in a total index size of 16 GB for the whole human genome NCBI HG18. Hobbes2 loads only the index into memory and the memory footprint of the index for HG18 is about 11 GB. Because Hobbes2 has a tight-knit multi-threaded framework that parallelizes both indexing and mapping, it took only a few minutes to build an index for HG18.

### Single end alignment on simulated data

We generated 100k simulated reads of length 100bp from HG18 using a read simulator, Mason [20]. We used the default profile setting of Mason with the illumina option. We used Rabema [21] benchmark to compare accuracies of read mappers. The benchmark was performed for an error rate 5%, or edit distance 5. To build a gold standard of simulated reads, we used RazerS3 in full-sensitive mode (we ran RazerS3 with its default setting for the performance comparison).

The benchmark found all, all of the best, and any of the best edit distance locations from the mapping results of each mapper. As the simulator generated original locations of simulated reads, we also measured the recall of each mapper, which is the fraction of reads whose original locations correctly reported.

Table 1 shows rabema scores in percentage, each of which is the average fraction of edit distance locations returned by a read mapper per read. Large numbers are total scores and small numbers are scores for reads with $\begin{array}{l} 0\;1\;2\\ 3\;4\;5 \end{array}$errors. We could not measure the mapping time of Masai with 16 threads since it does not support multi-threading. We omitted the mapping time of Bowtie2 with a single thread since it could finish only about 10% of 100k reads in 6 hours.

**Table 1:** Rabema benchmark results of mapping simulated 100k reads of length 100bp against HG18

	**Time (min:sec)**			**Benchmark category**																**Peak**
**Mapper**	**1 thr**	**16 thrs**		**All**				**All-best**				**Any-best**				**Recall**				**memory**
Hobbes2	9:43	1:33		99.85	100.0	100.0	100.0	99.99	100.0	100.0	100.0	99.99	100.0	100.0	100.0	98.97	100.0	99.90	99.68	14.6 GB
					99.99	99.94	97.48		100.0	100.0	99.84		100.0	100.0	99.84		99.34	99.04	99.77	
Hobbes	19:36	3:35		98.34	99.29	99.28	98.93	98.67	98.86	99.02	99.00	98.99	99.19	99.31	99.34	96.91	98.66	97.99	96.68	20.7 GB
					97.40	93.78	87.84		98.19	92.85	89.21		98.55	93.25	90.14		95.33	91.68	90.17	
Masai	18:11	−		99.83	100.0	100.0	100.0	99.94	100.0	100.0	100.0	99.94	100.0	100.0	100.0	99.03	100.0	100.0	100.0	16.9 GB
					99.73	99.18	97.69		99.69	98.73	98.52		99.69	98.73	98.52		99.71	98.77	98.56	
RazerS3	60:06	42:07		99.90	100.0	100.0	100.0	99.99	100.0	100.0	100.0	99.99	100.0	100.0	100.0	99.09	100.0	100.0	100.0	4.5 GB
					100.0	99.86	98.44		100.0	100.0	99.92		100.0	100.0	99.92		100.0	100.0	99.92	
Bowtie2	−	266:21		99.74	100.0	100.0	100.0	99.97	100.0	100.0	100.0	99.97	100.0	100.0	100.0	98.80	100.0	99.70	99.40	37.7 GB
					100.0	99.55	95.75		100.0	99.70	98.35		100.0	99.72	98.45		99.10	98.70	98.50	
BWA	75:04	12:20		97.73	100.0	99.98	99.64	98.89	100.0	99.98	99.61	98.90	100.0	99.98	99.61	97.91	100.0	99.98	99.45	4.8 GB
					93.47	82.91	75.15		93.03	78.87	70.57		93.03	78.98	70.73		92.47	78.55	71.18	
GEM	5:19	2:56		97.74	100.0	99.99	99.84	99.86	100.0	99.88	99.81	99.92	100.0	99.96	99.93	98.66	100.0	99.42	99.12	4.3 GB
					97.36	88.78	68.31		99.47	99.28	97.34		99.69	99.61	97.67		98.17	98.29	98.64	
Bowtie2*	0:31	0:32		91.34	98.87	97.75	93.55	97.08	97.65	97.33	95.69	99.29	100.0	99.45	97.65	95.96	97.75	96.88	95.00	3.2 GB
					81.07	53.90	21.95		95.38	93.98	93.74		97.41	96.24	95.89		94.60	93.33	93.95	
BWA*	2:08	0:25		92.27	100.0	99.82	96.90	98.79	100.0	99.83	99.41	98.83	100.0	99.89	99.49	97.31	100.0	99.17	97.76	4.5 GB
					79.11	45.49	16.99		92.57	78.26	70.34		92.70	78.60	70.73		90.39	77.11	70.35	
GEM*	0:31	0:13		94.48	100.0	99.38	97.61	99.86	100.0	99.88	99.81	99.92	100.0	99.95	99.92	98.62	100.0	99.28	99.06	4.3 GB
					90.10	69.11	35.34		99.41	99.17	97.37		99.72	99.61	97.75		98.24	98.35	98.94	

all: all mappings within the given edit distance threshold; all-best: all best mappings (i.e., all mappings with lowest edit distances); any-best: any best mappings (i.e., any mapping with lowest edit distances).

In terms of the accuracy of all mapping, the top three performers were RaserS3, Hobbes2 and Masai, with an accuracy score of 99.90, 99.85 and 99.83, respectively. Hobbes2 was slightly worse than RaserS3 on reads with high error rates. However, in terms of mapping time, Hobbes2 was much faster than both RaserS3 and Masai - six times faster than RaserS3 and twice as faster than Masai on a single thread, and 20 times faster than RaserS3 on 16 threads (there is no implementation of multi-threading on Masai.) BWA and Bowtie2, two most popular read mappers, trailed behind Hobbes2 in both accuracy and mapping time. GEM was faster than Hobbes2 on a single thread but slower than Hobbes2 on 16 threads. Although GEM mapped reads fast but it lost a lot of mapping locations of edit distance 4 and 5 and exhibited poor accuracy.

We also ran BWA, Bowtie2, and GEM in their default mode (or best mapping mode) to compare the performance. The results are reported at the end of Table 1 with mapper names, Bowtie2*, BWA*, and GEM*, respectively. Although they could produce results quickly, they exhibited poor mapping results. In particular, they lost most of locations of edit distance 5 and about a half of locations of edit distance 4.

### Single end alignment on real data

We used the human genome with HG18, caenorhabditis elegans (WormBase WS201), and drosophila melanogaster (FlyBase release 5.42) as reference sequences. For the human genome, we used the 100bp reads from specimen HG00096 of the 1000 genome project [22]. We also used 100bp reads taken from the DNA Data Bank of Japan (DDBJ) repository [23] with entry SRX026594 for the worm genome and SRX148416 for the fly genome.

Table 2 lists the experimental results of mapping 1 million reads of length 100bp against the human genome. We excluded Bowtie2 and BWA in the experiment since they are not designed as all mappers and exhibited poor mapping speed when aligning long, repetitive genomes. Hobbes2 mapped more reads than other read mappers while running significantly faster. Hobbes2 with 16 threads was about 3 times faster than Hobbes, 9 times faster than Masai and 42 times faster than RazerS3. By comparing the results of 500k reads and 1 million reads, we observed that the mapping time of each read mapper was approximately proportional to the number of input reads.

**Table 2:** Results of mapping 500k and 1 million single end reads of length 100bp against HG18

	**500,000 reads**						**1,000,000 reads**				
	**Read**	**# mappings**	**Time (min:sec)**		**Peak**		**Read**	**# mappings**	**Time (min:sec)**		**Peak**
**Mapper**	**mapped**	**(million)**	**1 thr**	**16 thrs**	**memory**		**mapped**	**(million)**	**1 thr**	**16 thrs**	**memory**
Hobbes2	91.476%	66.34	44:54	05:17	14.7 GB		91.558%	132.87	87:27	09:04	14.7 GB
Hobbes	91.449%	66.93	84:38	13:10	21.5 GB		91.533%	134.14	169:50	26:33	22.8 GB
Masai	91.473%	66.44	47:38	−	17.1 GB		91.555%	133.09	82:46	−	17.3 GB
RazerS3	91.472%	66.10	276:00	193:19	10.8 GB		91.554%	132.45	540:35	378:18	18.8 GB

The memory footprint of Hobbes2 was the smallest among the four mappers being compared on the dataset with 1 million reads. The memory requirement of Hobbes2 was independent of the number of input reads because it did alignment read by read. Masai used slightly more memory as the number of reads increased. The memory consumption of RazerS3 was greatly affected by the number of reads and the total number of mappings.

Table 3 shows the results of mapping 1 million reads of length 100bp against the C. elegans and D. melanogaster data sets. Hobbes had much higher memory footprint than Hobbes2 on the D. melanogaster data set. RazerS3 was unable to map reads on the D. melanogaster data set because it used too much memory space and was killed before the job was finished.

**Table 3:** Results of mapping 1 million single end reads of length 100bp against C. elegans and D. melanogaster

	**C. elegans**						**D. melanogaster**				
	**Read**	**# mappings**	**Time (min:sec)**		**Peak**		**Read**	**# mappings**	**Time (min:sec)**		**Peak**
**Mapper**	**mapped**	**(million)**	**1 thr**	**16 thrs**	**memory**		**mapped**	**(million)**	**1 thr**	**16 thrs**	**memory**
Hobbes2	91.003%	5.71	03:09	00:41	0.8 GB		95.470%	438.33	79:35	28:46	1.2 GB
Hobbes	90.994%	5.84	04:26	01:06	1.4 GB		95.436%	453.43	90:11	57:01	29.1 GB
Masai	91.002%	5.68	05:19	−	0.9 GB		95.466%	446.98	131:11	−	1.3 GB
RazerS3	91.002%	5.69	13:28	12:35	1.4 GB		−	−	−	−	96.5 GB

Again, Hobbes2 mapped more reads than other mappers for both data sets. For the C. elegans data set, Hobbes2 with 16 threads was about twice as faster as Hobbes, 7 times faster than Masai and 18 times faster than RazerS3. For the D. melanogaster data set, Hobbes2 also exhibited the best mapping speed in both single threaded and multi-threaded cases. Hobbes2 used the least amount of memory in both data sets.

The significant improvement of Hobbes2 is mainly due to our improved method of generating initial candidates. It is very important to quickly generate a small number of candidates in edit distance case since most of the mapping time is spent in the verification process, which requires a more expensive dynamic programming procedure. Table 4 shows the number of candidates initially generated by the read mappers and the time for generating candidates. Hobbes2 generated the least number of candidates among the read mappers. Hobbes generated candidates very fast with the help of bit vectors, but Hobbes2 and Masai generated about four times fewer candidates than Hobbes. Hobbes2 produced candidates more than two times faster than Masai while generating fewer candidates.

**Table 4:** Filtration of 500k reads of length 100bp on HG18

**Mapper**	**Filtration time (min:sec)**	**Number of candidates**
Hobbes2	04:14	1,161,828,591
Hobbes	01:45	3,833,554,010
Masai	09:48	1,190,600,997
RazerS3	15:01	7,007,527,711

### Paired end alignment

For paired end read alignment, we used the human genome HG18 as the reference sequence. We ran experiments using 100bp read pairs from specimen HG00096.

Our performance results for the paired end alignment are summarized in Table 5. We excluded BWA in the experiment since it does not support the minimum insert size. Bowtie2 in all mapping mode could not finish the mapping in 24 hours. We used Bowtie2 in the default mode, which is listed as Bowtie2* in Table 5. Since Masai does not directly support mapping paired end reads, we separately ran masai_mapper for each read file to output results in Masai’s raw format, and merged the results using masai_output_pe to produce mappings in the SAM format.

**Table 5:** Results of mapping 1 million × 2 paired end reads of length 100bp against HG18

	**Read**	**Mapping time (min:sec)**		**Peak**
**Mapper**	**mapped**	**1 thr**	**16 thrs**	**memory**
Hobbes2	86.66%	59:40	11:12	14.9 GB
Hobbes	86.52%	61:54	24:43	20.4 GB
Masai	84.07%	68:46	−	17.3 GB
RazerS3	86.68%	420:07	342:14	17.5 GB
Bowtie2*	82.12%	8:40	0:52	3.6 GB

We observed that Hobbes2 was the fastest among all mappers in both single threaded and multi-threaded cases. With 16 threads, Hobbes2 was about twice as faster as Hobbes, and 31 times faster than RazerS3. In terms of mapped pairs, Hobbes2 was similar to Hobbes and RazerS3, but was better than Masai. Hobbes used the least amount of memory for the paired end mapping. Although Bowtie2* ran very fast, it lost many mapping pairs, and thus exhibited poor mapping quality compared with other all mappers.

## Conclusion

Hobbes2 efficiently finds all mapping locations of a read in a reference genome. We have shown that Hobbes2 is substantially faster than state-of-the-art all mappers while maintaining similar accuracy. In addition, Hobbes2 consumes less memory space than other read mappers for long reads since it does not rely on additional data structures other than inverted lists of *q*-gram signatures.

Our experiments have also shown that Hobbes2 scales very well in multi-threaded environment, and exhibits the best performance among the competitors. Given today’s trend toward massively multi-core CPUs, read mappers with good multi-thread support will likely become more necessary in the future.

Because of its simplicity, we believe the candidate generation method implemented in Hobbes2 can also be adapted for other read mapping programs for improving their performance.

## Competing interests

The authors declare that they have no competing interests.

## Authors’ contributions

JK developed the filtering technique using additional prefix grams and CL applied the technique to the read alignment problem. JK implemented the read mapping algorithm using the technique and ran the experiments. XX designed experiments and analyzed the results. JK wrote the manuscript, and CL and XX read and edited the manuscript. All authors read and approved the final manuscript.

## Supplementary Material

Additional file 1 **Supplementary material.** This file contains supplementary text, algorithm, figures, and tables. Click here for file
