# Supplementary material for: Improving read mapping using additional prefix grams
Source: BMC Bioinformatics. 2014 Feb 5;15:42. doi: 10.1186/1471-2105-15-42 (PMC3927682; doi:10.1186/1471-2105-15-42)
Supplement: Additional file 1 — Supplementary material. This file contains supplementary text, algorithm, figures, and tables. [file 1471-2105-15-42-S1.pdf]

Supplementary Material for "Improving Read Mapping Using Additional Prefix Grams"

S1 Candidate Generation Algorithm

Algorithm 1: GenerateCandidates(L, k, Δ_{gap})

input : L is a set of inverted lists,
k is a distance threshold,
Δ_{gap} is an allowed gap between two matched q-gram (it is set to 0 for hamming distance and set to k for edit distance).

output: A set of candidate locations C.

```
sort inverted list in L by their length;
foreach element loc ∈ L[1] do  C.push_back(loc − L[1].gramloc, 1);
for p ← 2 to k + 2 do
    C_new ← empty set of (location,count) pairs;
    i ← 1;
    j ← 1;
    carry ← 0;              // deferred increment of count
    /* assume each list contains max value at the end */
    while i ≤ C.size() or j ≤ L[p].size() do
        candidate_loc ← L[p][j] − L[p].gramloc;      // normalize L[p][j] to find a candidate location
        if C[i].location − candidate_loc > Δ_gap then
            C_new.push_back(candidate_loc, 1 + carry);
            j ← j + 1;
            carry ← 0;
        else if C[i].location − candidate_loc < Δ_gap then
            C_new.push_back(C[i].location, C[i].count + carry);
            i ← i + 1;
            carry ← 0;
        else
            if C[i].location = candidate_loc then
                C_new.push_back(C[i].location, C[i].count + 1);
                i ← i + 1;
                j ← j + 1;
                carry ← 0;
            else if C[i].location > candidate_loc then
                C_new.push_back(candidate_loc, 2);
                j ← j + 1;
                carry ← 1;          // set deferred increment of C[i].count
            else
                C_new.push_back(C[i].location, C[i].count + 1);
                i ← i + 1;
                carry ← 1;          // set deferred increment of the count of candidate_loc
    C ← C_new;
remove every element from C whose count is less than 2;
Return C;
```

S2 Additional Experiments

Effect of the gram length: we ran an experiment to figure out the effect of the gram length, q, on the performance. We also compared the $k + 1$ prefix gram technique with the $k + 2$ prefix gram technique, where k is an edit distance threshold. In the experiment, we mapped 500k 100 bp single-end reads against HG18 using 16 threads for an edit distance threshold 5. Figure S1 shows the results. In $k + 1$ prefix gram technique, the performance of read mapping improved until $q = 13$. However, the performance degraded when $q = 14$. The $k + 2$ prefix gram technique exhibited the best performance when $q = 11$. If we use large q, we reduce sizes of inverted lists and as a result we generate fewer candidates. As we increase q size, however, the number of different grams grows sharply and we need much time to look up an inverted index to find an inverted list of each gram. Increasing q size also affects selecting good grams.

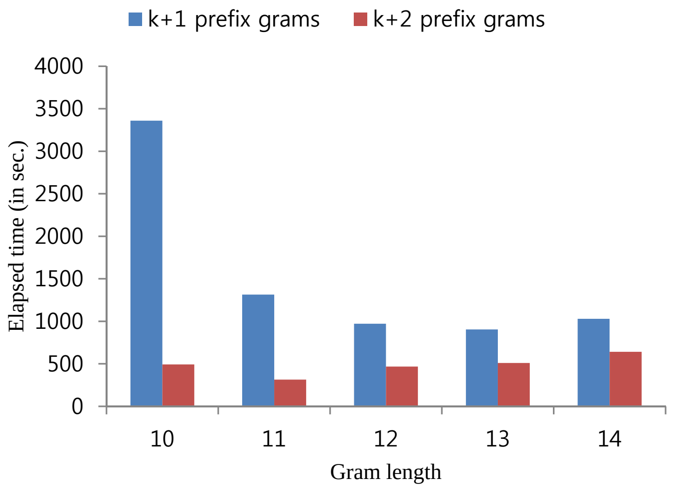

Figure S1: Performance results according to different gram lengths

Effect of the number of additional prefix grams: Figure S2 shows mapping times according to the number of additional prefix q-grams. For the experiment, we mapped 500k 100 bp single-end reads against HG18 using 16 threads for an edit distance threshold 5. To select more than one additional q-grams, we simply used the same heuristic described in the paper. As depicted in the figure, only the first additional prefix gram brought a significant improvement. The second and third prefix resulted in marginal improvements only.

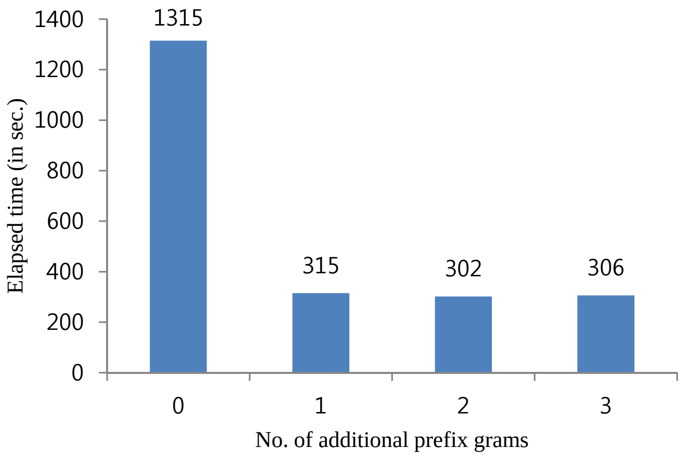

Figure S2: Performance results according to different additional prefix grams

Experiments with hamming distance constraints: since Hobbes2 runs exactly the same as Hobbes for short reads, we conducted experiments only on 76bp and 100bp reads of HG18 using a single thread for Hamming distance constraints. 76bp reads taken from DDBJ repository with entry DRX000360. Table S1 shows the fraction of reads with at least one mapping, the mapping time, and the peak memory consumption. We ran tools using a single thread in the experiments. Hobbes2 was slightly slower than Hobbes with 32 bit vectors but it was faster than Hobbes with 16 bit vectors. Hobbes2 was 3 to 6 times faster than Masai, GEM, and RazerS3. In terms of memory consumption, Hobbes2 required only 14.8 GB, which was about a half of the memory footprint of Hobbes with 32 bit vectors. GEM lost only a small amount of mappings for 76bp reads, but lost many mappings for 100bp reads. RazerS3 did not correctly map both single end and paired end reads.

Table S1: Results of mapping 500k single-end reads and 500k × 2 paired end reads against HG18

	single end, 76bp, 3 errors			single end, 100bp, 5 errors			paired end, 100bp, 5 errors		
mapper	read mapped (%)	total mappings (million)	mapping time (min:sec)	read mapped (%)	total mappings (million)	mapping time (min:sec)	read mapped (%)	mapping time (min:sec)	peak memory
Hobbes2	91.43	73.37	03:35	90.10	60.39	05:07	84.39	09:24	14.8GB
Hobbes-16bit	91.43	73.37	04:29	90.10	60.39	08:51	84.39	09:08	21.5GB
Hobbes-32bit	91.43	73.37	02:49	90.10	60.39	04:58	84.39	08:16	27.1GB
Masai	91.43	73.37	12:50	90.10	60.39	14:09	81.96	25:23	18.2GB
GEM	91.43	73.37	19:58	85.23	45.62	23:28	79.70	37:28	10.4GB
RazerS3	67.57	4.33	13:54	64.67	1.47	34:07	50.52	63:08	7.6GB

Experiments in m mapping mode: Hobbes2 supports m mapping mode, which reports only those reads such that the number of total mapping locations is less than or equal to a given threshold m. We ran experiments to evaluate Hobbes2 in m mapping mode. We mapped 500k 100 bp single-end reads against HG18 using 16 threads for an edit distance threshold 5. Table S2 shows the results. If m is very large, the mapping time is similar to that of all mapping mode (e.g. $m = 10000$ in Table S2). However, for a reasonable m value (up to 1000 in Table S2), m mapping mode is 1.5 to 2 times faster than all mapping mode while losing only a small set of reads.

Table S2: Results of mapping 500k single-end reads against HG18 in m mapping mode

threshold (m)	mapped reads (%)	mapping time (min:sec)	peak memory
1	82.021	2:44	14.7G
10	87.222	2:57	14.7G
100	88.993	3:16	14.7G
1000	89.877	3:48	14.7G
10000	91.458	4:49	14.7G
all	91.476	5:17	14.7G

S3 Configuration of Each Tool

We configured read mappers used in our experiments as follows.

Hobbes2. We used Hobbes version 2.1. We built indices with 16 bit vectors for both edit distance and Hamming distance constraints. In order to use Hobbes2 as an all mapper we specified `-a` option. We used `--hamming` option for Hamming distance constraints and `--indel` option for edit distance constraints. For the paired end mapping, we set `--min` and `--max` parameters to 110 and 290 respectively. For multi-threaded execution, we used `-p` option. Note that Hobbes2 generates output in the SAM format with cigar strings by default.

Hobbes. Version 1.5 was used. We used 16 bit vector inverted index for edit distance constraints. Since Hobbes does not calculate cigar strings by default, we used `--cigar` option to produce output with cigar strings. Other configurations of Hobbes were the same as Hobbes2.

RazerS3. Version 3.2 was used. To use RazerS3 as an all mapper, we used set the parameter `-m` to 1,000,000. To measure the total number of mappings, we manually removed duplicated outputs. We do not include the time for duplication eliminations. To output results in the SAM format, we used the extension ".sam" in output files. For multi-threaded execution, we used `-tc` option. We set the parameters `-ll` and `-le` to 200 and 90 respectively for the paired end mapping. For Hamming distance constraints, we used `--no-gaps` option and set the parameter `-rr` to 100 to produce all possible mappings. We used the default value of the parameter `-rr` in edit distance case. For other options, we used the default values of RazerS3.

Masai. Version 0.7.1 was used. To use Masai as an all mapper, we used '`-mm all`' option. We set `-ll` and `-le` parameters to 200 and 90 respectively for the paired end read mapping. In Hamming distance case, we specified `--no-gaps` option. We used the extension ".sam" in output files to output results in the SAM format. To produce results for paired end reads, we converted Masai's raw outputs into SAM format outputs by running the tools `masai_output_pe` separately. We included the conversion time in the mapping time.

BWA. Version 0.7.4 was used. We used the `-N` option to configure BWA as an all mapper. To specify an edit distance 5, we set `-n`, `-o`, and `-e` parameters to 5, 5, and -1, respectively. For multi-threaded execution, we used `-t` option. We used the default values for other options.

Bowtie2. Version 2.1.0 was used. We used the `-a` option to configure Bowtie2 as an all mapper. To specify an edit distance 5, we used `--end-to-end` and `--ignore-quals` options and set `--mp`, `--np`, `--rdg`, `--rfg`, and `--score-min` parameters to [1,1], 1, [0,1], [0,1], and [L,0,−0.05], respectively. For multi-threaded execution, we used `-p` option. We used the default values for other options. We set `-min` and `-max` paramters to 210 and 390 respectively for paired end reads.

GEM. Version 1.376 was used. To specify an edit distance 5, we used '`quality-format = ignore`' and set both `-m` and `-e` parameters to 5. To configure GEM as an all mapper, we set `-d` paramter to `all` and `-s` paramter to 1,000,000. We converted outputs of `gem-mapper` into SAM format outputs using `gem-2-sam`. We included the conversion time in the mapping time.
